# The paediatric hepatic international tumour trial (PHITT): clinical trial design in rare disease

**DOI:** 10.1186/1745-6215-16-S2-P224

**Published:** 2015-11-16

**Authors:** Veronica Moroz, Bruce Morland, Gregory Tiao, Eiso Hiyama, Pamela Kearns, Keith Wheatley

**Affiliations:** 1 University of Birmingham, Birmingham, UK; 2 Birmingham Children’s Hospital NHS Trust, Birmingham, UK; 3 UC Department of Surgery, Cincinnati, USA; 4 Hiroshima University Hospital, Hiroshima, Japan

## 

Hepatoblastoma is a comparatively uncommon paediatric solid tumour, while hepatocellular carcinoma is very rare.

The Paediatric Hepatic International Tumour Trial will be the single largest clinical trial undertaken in paediatric liver cancer patients as collaboration between the European Study Group for Paediatric Liver Tumours, Children’s Oncology Group and Japanese Study Group for Pediatric Liver Tumors.

In the context of the low incidence paediatric cancer, an overarching study with therapeutic questions for the separate tumour types and risk groups will be more efficient than setting up separate studies for each group. Hence, the design will incorporate randomised comparisons for five different patient groups. It is difficult to obtain feasible sample sizes based on conventional frequentist design criteria. Therefore, Bayesian methods based on probability distributions will be used with event-free survival as the primary outcome measure for each comparison summarised by hazard ratio. Posterior probability distributions, with non-informative priors, based on the number of events will be used to give probabilities of one treatment being better than another. Operating characteristics are explored through simulations of various scenarios given pre-specified decision criteria. The level of evidence needed will depend on the clinical question: for example, a stronger level of evidence is likely to be needed if treatment is being intensified than if two regimens of comparable intensity are being compared.

It is important to investigate promising approaches in randomised trials. The design will enable quicker and more efficient identification of effective therapies, leading to improved outcomes for children with liver cancer.

